# Study of resilience in a population of tunisian residents and interns in medicine

**DOI:** 10.1192/j.eurpsy.2022.1597

**Published:** 2022-09-01

**Authors:** A. Zouari, F. Guermazi, F. Tabib, A. Guermazi, S. Hentati, I. Baati, J. Masmoudi

**Affiliations:** HEDI CHAKER HOSPITAL, Psychiatry A, sfax, Tunisia

**Keywords:** resident, medical, resilience, intern

## Abstract

**Introduction:**

Resilience is the ability to bounce back or cope successfully with stress. Fostering resilience is a promising way to mitigate the negative effects of stressors and prevent burnout.

**Objectives:**

Study the level of resilience among Tunisian medical interns and residents.

**Methods:**

We conducted a cross-sectional descriptive study between March 1 and April 15, 2021. Medical interns and residents were invited to complete an online self-questionnaire. We used the Brief Resilience Scale (BRS) to assess the level of resilience.

**Results:**

The total number of participants was 56 of which 28.6% were male. The average age was 26.76±2.52 years. Most of the students had studied at the Faculty of Medicine in Sfax, 58.9%. 64.3% of the participants were residents, 55.4% of them in a medical specialty. 75% of participants were working in a medical department. The average years of practice was 2.27±1.23. The number of working hours per week exceeding 40 hours was found in 60.7% of participants. The number of shifts per month exceeding 4 shifts was found in 67.9%. 46.4% of the participants wanted to change their profession and 44.6% regretted choosing medicine. The mean score by BRS was 2.79±0.48. The level of resilience was high in 42.9% of the participants and normal in the rest of the respondents.

**Conclusions:**

The level of resilience was normal to high in Tunisian medical interns and residents. Assessing the presence of burnout and the coping strategies used could provide insight into the quality of work life.

**Disclosure:**

No significant relationships.

